# The Role of the Liver in the Pathophysiology of PCOS: A Literature Review

**DOI:** 10.3390/biom15010051

**Published:** 2025-01-02

**Authors:** Abrar Alhermi, Heather Perks, Varsha Nigi, Noor Altahoo, Stephen L. Atkin, Alexandra E. Butler

**Affiliations:** 1 School of Medicine, Royal College of Surgeons of Ireland, Busaiteen, Adliya P.O. Box 15503, Bahrain; 20205606@rcsi-mub.com (A.A.); 23204151@rcsi-mub.com (H.P.); 23204032@rcsi-mub.com (V.N.); 20204236@rcsi-mub.com (N.A.); 2 Research Department, Royal College of Surgeons of Ireland, Busaiteen, Adliya P.O. Box 15503, Bahrain; satkin@rcsi.com

**Keywords:** polycystic ovary syndrome (PCOS), insulin resistance, nonalcoholic fatty liver disease (NAFLD), hepatokines, non-coding RNA

## Abstract

Polycystic ovary syndrome (PCOS) is the most common endocrine metabolic disorder found in women of reproductive age and is characterized by both metabolic and reproductive dysfunction. Women with PCOS commonly have insulin resistance, increased susceptibility to type 2 diabetes mellitus, dyslipidemia, hyperinsulinemia, increased cardiovascular risk, hepatic steatosis, infertility, and an overall reduction in physical and psychological well-being. Several previous studies have shown a causal association between PCOS and hepatic disorders, such as chronic liver disease (CLD) and nonalcoholic fatty liver disease (NAFLD), where PCOS was identified as contributing to the hepatic features. Whilst it is recognized that PCOS may contribute to hepatic dysfunction, there is also evidence that the liver may contribute to the features of PCOS. The purpose of this review is to discuss the current understanding regarding hepatic involvement in PCOS pathophysiology, the inflammatory markers and hepatokines involved in the development of PCOS, and the role of genetics in the occurrence of PCOS. This review illustrates that PCOS and NAFLD are both common disorders and that there is both genetic and metabolic linkage between the disorders. As such, whilst PCOS may contribute to NAFLD development, the converse may also be the case, with a potential bidirectional relationship between PCOS and liver disease.

## 1. Introduction

Polycystic ovary syndrome (PCOS) is a multifaceted endocrine and metabolic disorder seen in pre-menopausal women [1]. Globally, it is estimated to affect between 5 and 20% of females of reproductive age [2]. PCOS is a heterogeneous disorder that presents diverse clinical manifestations, has an uncertain etiology, and has an undetermined pathophysiology [3]. It is defined by internationally accepted signs and symptoms comprising polycystic ovaries, androgen excess, and ovarian dysfunction [4]. Common clinical signs of hyperandrogenism in females are alopecia, acne, and hirsutism [5]. The majority of women with PCOS experience metabolic dysfunction [6], such as insulin resistance, which is present in up to 80% of women with PCOS [7]. Women with PCOS also have a higher risk of developing type 2 diabetes mellitus, hyperinsulinemia, hypertension, dyslipidemia, cerebrovascular and cardiovascular events, hepatic steatosis, infertility, mood disorders, and an impaired quality of life [8]; therefore, PCOS is associated with significant healthcare and economic costs and resource utilization [9].

Several diagnostic criteria exist to define PCOS; these include the 2003 Rotterdam criteria, the 1990 National Institutes of Health criteria (NIH), and the 2006 Androgen Excess Society (AES) criteria [10]. Current literature highlights the frequent utilization of the Rotterdam criteria, as clinicians prefer the broad categories [11]. The Rotterdam criteria for PCOS require two of the following three characteristics to be present: oligo- or anovulation, clinical and/or biochemical evidence of hyperandrogenism, and polycystic ovarian morphology, which is defined as having an ovarian volume > 10 mL in either ovary and/or the presence of ≥12 follicles of 2–9 mm in diameter [12]. As of 2023, the revised Rotterdam criteria are utilized for diagnosing PCOS. This diagnosis requires at least two of the following three characteristics to be present: biochemical or clinical hyperandrogenism, ovulatory dysfunction, and/or polycystic ovaries on ultrasound or elevated anti-Müllerian hormone (AMH) levels [13]. Following the establishment of the criteria, four main phenotypes of PCOS were recognized: phenotype A (oligo–anovulation, hyperandrogenism, and polycystic ovaries), phenotype B (oligo–anovulation and hyperandrogenism), phenotype C (polycystic ovaries and hyperandrogenism), and phenotype D (polycystic ovaries and oligo–anovulation) [14]. Phenotypes A and B are the most common and classical phenotypes, and where insulin resistance is present, this is followed by phenotype C, ovulatory PCOS, and phenotype D, described as non-hyperandrogenic PCOS [11]. The NIH criteria require oligo- or chronic anovulation and clinical and/or biochemical signs of hyperandrogenism. By contrast, the AES criteria require the presence of both clinical and/or biochemical evidence of hyperandrogenism and either polycystic ovarian morphology and/or oligo–anovulation for the diagnosis of PCOS [15]. Thus, the NIH criteria correspond to phenotypes A or B, while the AES criteria correspond to phenotypes A, B, and C [10]. The etiology of PCOS is not completely understood. However, increasing evidence suggests that PCOS is a multi-genetic disorder with environmental and epigenetic influences [16,17], including factors such as diet and lifestyle [18]. To date, three principal components that contribute to the pathophysiology of PCOS have been identified [1]. The first is high familial heritability and aggregation, as exemplified by a recent study conducted in 2019 reporting that there is a five-fold higher risk of developing PCOS in daughters born to women with PCOS in comparison to those born to women without PCOS [19]. However, the mode of inheritance is unknown and is suggested to be multifactorial and polygenic in nature [2]. Second is the contribution of environmental factors, such as fetal exposure prenatally to the intrauterine environment of mothers with PCOS and the lifestyle following birth [3,10]. Lastly, there is the interaction between metabolic disorders and reproductive dysfunction, where during the development of PCOS, the presence of insulin resistance and hyperandrogenism causes them to amplify one another, and this is also affected by a defective hypothalamic–pituitary–ovarian axis [10,20].

Despite the existence of several theories regarding the pathophysiology of PCOS, the mechanisms that contribute to the common systemic manifestations, including insulin resistance and hyperandrogenism, are still unclear. Previous literature has indicated an association between PCOS and liver disease, which has become an area of active research.

Worldwide, chronic liver disease (CLD) is a leading cause of morbidity and mortality, representing the 15th leading cause of morbidity and the 11th leading cause of death [19].

An increasing prevalence of liver disease, including nonalcoholic fatty liver disease (NAFLD), has been reported in patients with PCOS. The key features of PCOS, including insulin resistance, obesity, and androgen excess, have been implicated as factors that contribute to the development of NAFLD in PCOS [21]. However, there is growing interest in exploring the possibility of a bidirectional relationship between liver disease and PCOS, where it has been proposed that both diseases exacerbate one another rather than one directly causing the other.

## 2. Methodology

The search was performed in PubMed, Medline, ScienceDirect, Embase, and Google Scholar from inception until July 2024 using the following keywords: polycystic ovary syndrome (PCOS), liver metabolism, insulin resistance, nonalcoholic fatty liver disease (NAFLD), oxidative stress, hepatokines, non-coding RNA, proprotein convertase subtilisin/kexin type 9 inhibitors (PCSK9i), cannabinoid receptor 1 (CNR1), steroid hormone binding globulin (SHBG), patatin-like phospholipase domain-containing 3 gene (PNPLA3), cytochrome P450 (CYP450), liver clock genes. Inclusion criteria for this research paper included articles in English only. References cited in the identified articles were individually examined for further reference. Exclusion criteria included articles published in a language other than English.

## 3. Association Between Liver Metabolism and PCOS Pathophysiology

### 3.1. Lipotoxicity and Insulin Resistance

Lipotoxicity, as a cause of fat accumulation in liver cells leading to ongoing cellular dysregulation [22], is a primary factor in the development of insulin resistance due to abnormal fat distribution and lipid deposition in various non-adipose tissues, thereby resulting in oxidative/endoplasmic reticulum stress, as well as inflammation [23].

Women with PCOS tend to have higher intra-abdominal fat deposition along with an increase in subcutaneous abdominal adipose tissue with enlarged adipocytes, which are known to promote lipotoxicity [24]. These effects were reported in a study performed on female mice where the administration of a high-fat diet led to ovarian cell lipotoxicity with reduced rates of ovulation and impaired ovum fertilization [24].

Lipotoxicity at the tissue level can be attributed to hyperplasia of white adipocytes along with infiltration of macrophages that induces the release of cytokines, adipokines, and proteins with chemo-attractant properties [25]. An increase in proinflammatory cytokines leads to reduced insulin sensitivity, primarily through modification of glucose transporters in women diagnosed with PCOS [25]. Furthermore, the excess uptake of free fatty acids into non-adipose cells aggravates the condition due to an impairment in the insulin signaling pathway through excessive phosphorylation of insulin receptor serine as well as disruption of mitochondrial oxidative phosphorylation [26].

Up to 80% of women diagnosed with PCOS have insulin resistance due to altered insulin receptors or post-receptor signaling, and abnormal secretion of adipokines, along with defective steroid metabolism [26]. A combination of these factors leads to hyperinsulinemia, which, along with hyperandrogenism, tends to worsen the PCOS phenotype [26]. Several factors result in adverse metabolic outcomes in PCOS patients, including androgen excess within adipose tissue/cells and dysregulated lipid metabolism [27,28]. Additionally, this study showed that androgen excess played a vital role in the creation of a lipotoxic metabolic environment, indicating a bidirectional relationship between lipotoxicity, the liver, and PCOS [27].

### 3.2. Nonalcoholic Fatty Liver Disease (NAFLD)

There is an association with increased abdominal fat in women with PCOS as a risk factor for the development of nonalcoholic fatty liver disease (NAFLD) accompanied by varying levels of fibrosis and inflammation [26].

A causal relationship between PCOS and NAFLD, and whether insulin resistance and abnormal levels of serum androgens contribute to the pathophysiology of these conditions, have not been extensively investigated. The lack of investigation is due to the confounding or reverse causation bias in conventional observational analyses [29]. One study conducted by Liu et al. used Mendelian randomization (MR) using germline genetic variants as variables to reduce the risk of bias [29]. The results from MR analyses found a unidirectional causal relation, genetically predicting that NAFLD increases the risk of PCOS development [29]. More recent studies reported that women diagnosed with PCOS tend to display more severe histological features such as nonalcoholic steatohepatitis (NASH), fibrosis, and cirrhosis and have a two-fold higher risk of developing NAFLD compared to the control groups [30].

Furthermore, other studies show that patients diagnosed with PCOS have a high prevalence of metabolic syndrome, putting them at risk for NAFLD [31,32]. In 15–30% of PCOS patients, fluctuations in alanine aminotransferase (ALT) and aspartate transaminase (AST) levels associated with NAFLD were seen [33]. These findings further suggest that there exists a bidirectional relationship between liver dysfunction, NAFLD, and PCOS.

From a nomenclature perspective, NAFLD has recently been transitioned to metabolic dysfunction-associated steatotic liver disease (MAFLD), while NASH has transitioned to metabolic dysfunction-associated steatohepatitis (MASH) [34]. These changes were instigated to decrease stigma, inspire researchers to understand the disease better, and promote successful treatment development [35].

### 3.3. Oxidative Stress

Oxidative stress (OS) is significantly associated with PCOS. OS is denoted by a disruption between oxidants and antioxidants, resulting in the overproduction of reactive oxygen species (ROS) [36]. Studies have demonstrated that PCOS patients have increased circulating oxidative stress markers such as homocysteine, malondialdehyde, and asymmetric dimethylarginine [37]. Free radical and non-free radical ROS include hydrogen peroxide (H_2_O_2_), superoxide, singlet oxygen, and hydroxyl radicals [38]. These oxygenated molecules have cytotoxic effects through free radical pathways and direct interaction with lipids, DNA, and proteins, overwhelming antioxidant defenses [39]. Studies have shown that the body’s antioxidant defense mechanism in PCOS patients is affected by a range of factors, including increased levels of DNA strand breakage, DNA damage by H_2_O_2_, and increased micronucleus (MN) frequency (a measure of genomic instability) as well as increased mitochondrial DNA (mt) copy number, leading to oxidative stress [38].

Additionally, PCOS patients have a higher antioxidative versus oxidative state, implying disruption of oxidants and antioxidant levels in PCOS patients [40]. Furthermore, a rise in ROS leads to impairment of the oxidative metabolism of mitochondria, insufficient energy supply, and increased oxidative stress. Consequently, this results in abnormal morphology and increasing apoptosis of granulosa cells (GCs) [36]. Studies have shown that women with PCOS tend to have higher mean ROS levels in their follicular fluid (FF), suggesting poorer oocyte quality and, thus, embryo quality [41].

The mechanism underlying the increased OS in PCOS patients involves the stimulation of various protein kinases, which results in the phosphorylation of serine/threonine on insulin receptor substrates. Phosphorylation will cause degradation and insulin sensitivity reduction, ultimately contributing to the development of insulin resistance (IR) [36]. The activation of serine kinases also leads to the production of a suppressor of cytokine signaling 3 (SOCS3), which inhibits the expression of IRS-1 and tyrosine phosphorylation, causing a decrease in the sensitivity of tissue cells to insulin, thus promoting IR [42]. Oxidative stress directly impacts the liver through the metabolic aberrations associated with PCOS, such as insulin resistance and obesity [37]. OS has also been associated with liver dysfunction and NAFLD, common in PCOS. OS development contributes to liver inflammation, fibrosis, and insulin resistance, all of which are interrelated with PCOS pathophysiology [43].

In the context of PCOS, oxidative stress plays a crucial role in the development of liver conditions, including NAFLD and liver cancer. Furthermore, obesity, insulin resistance, and increased levels of free testosterone that are seen in PCOS patients exacerbate oxidative stress, a risk factor for liver disease [38]. Moreover, the increased levels of ROS in PCOS lead to cellular damage in hepatocytes and induce oxidative stress, hence contributing to insulin resistance and liver disease. However, this may be obesity-dependent [44,45]. Studies have demonstrated that oxidative stress affects the liver by promoting lipid peroxidation and damage to lipids, proteins, and DNA compounds, all of which contribute to inflammatory protein production and stress-related protein kinase activation, promoting insulin resistance. Additionally, the use of resveratrol, an antioxidant agent, reduced the production of free radicals, peroxidation of lipids, and oxidative stress in the livers of study rats with PCOS induced by subcutaneous injection of androgens [46].

### 3.4. Chronic Inflammation

Chronic low-grade inflammation and elevation of various proinflammatory cytokines, including C-reactive protein, are present in PCOS (Table 1) [18,47]. Studies have found a higher incidence of elevated CRP levels in PCOS patients with insulin resistance and obesity [48]. CRP is produced in the liver and has been shown to be produced in response to inflammation, specifically by interleukin-6 (IL-6) and tumor necrosis factor-alpha (TNF-a) [49,50]. Increased CRP levels could be due to a single-nucleotide polymorphism (SNP) in the proinflammatory cytokines in association with PCOS, indicating a genotype-specific predisposition to PCOS [47]. However, the underlying mechanism has yet to be established. Studies have suggested an association between CRP and insulin resistance, thus increasing cardiovascular risk. Therefore, CRP could be used as a biomarker to assess cardiovascular risk and the efficacy of novel therapeutic targets [50]. Also, studies have shown that increased advanced glycation end products (AGEs) and OS present in PCOS patients impact the liver [49]. A study of female rat exposure to dihydrotestosterone (DHT) demonstrated that DHT led to obesity, insulin resistance, hepatic steatosis, lipid deposition, and various degrees of hepatic inflammation. Additionally, the study showed NAFLD development in DHT-exposed rats through mitochondrial dysfunction, oxidative stress, and an imbalance in apoptosis and autophagy [51]. Chronic inflammation in PCOS patients, recognized by the presence of increased inflammatory markers and further dysregulated by hyperandrogenemia [52], contributes to dysfunction of the liver and disturbances in metabolism and may worsen NAFLD and insulin resistance [53].

Furthermore, in PCOS patients, inflammatory markers such as TNF-a have shown direct effects on the liver, highlighting the association between chronic inflammation in PCOS patients and liver dysfunction. Additionally, research has indicated the association between chronic inflammation and the liver by the effect of curcumin, an anti-inflammatory agent, in decreasing oxidative stress and preventing diabetic complications [54].

**Table 1:** The effect of inflammatory and other key markers on the development of PCOS.

Inflammatory Markers	Role in PCOS	References
C-reactive protein	Elevated potentially due to SNPs in proinflammatory cytokines associated with PCOS. Elevated levels result in chronic low-grade inflammation. Higher incidence in phenotypes with insulin resistance and obesity, thereby increasing the risk of cardiovascular complications.	[18,47,50]
Interleukin-6	Elevated in PCOS. Major proinflammatory cytokine in low-grade chronic inflammation. Close association with cardiovascular diseases in women with PCOS through various metabolic, endothelial, and coagulant interactions.	[49,55]
Tumor necrosis factor-alpha	Elevated in PCOS. Increases insulin resistance, promotes hyperandrogenism, and negatively impacts follicular development.	[54,56]
Dihydrotestosterone	Elevated in PCOS. Increased predisposition to obesity, inflammation, insulin resistance, and hepatic steatosis. Mitochondrial dysfunction leads to increased oxidative stress and imbalance in apoptosis and autophagy.	[51,57]
Advanced glycation end products	Altered levels in PCOS. Interaction with membrane receptors results in oxidative stress along with inflammation, hyperandrogenism, insulin resistance, and ovulatory dysfunction.	[58]

## 4. Association of Hepatokines in the Pathophysiology of PCOS

Recent research has highlighted the potential role of hepatokines in the pathogenesis and metabolic complications associated with PCOS. Hepatokines have emerged as crucial mediators of liver dysfunction, insulin resistance, and the hormonal imbalances seen in PCOS.

### 4.1. Fetuin-A

**Figure 1:**
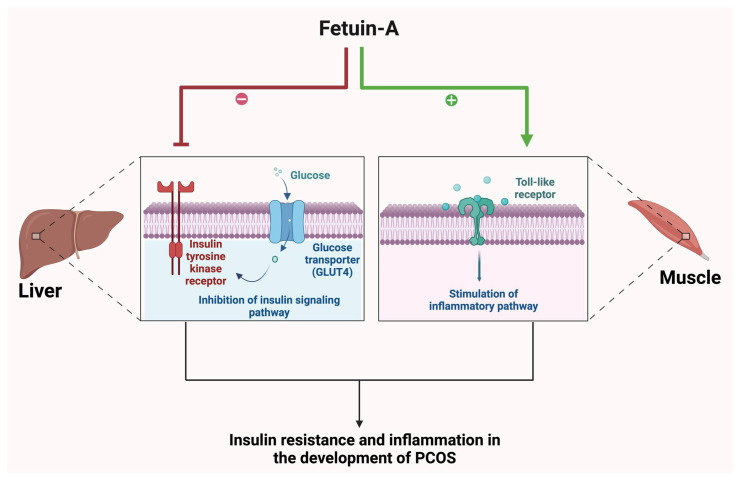
The role of fetuin-A in the progression of PCOS. Schematic illustrating inhibition of the insulin signaling pathway in the liver and the activation of the inflammatory pathway in muscle by fetuin-A. Inflammation contributes to the development of PCOS. In contrast, disruption of the insulin signaling pathway leads to insulin resistance, which also plays a significant role in the progression of the condition.

Fetuin-A is a multifaceted hepatokine, functioning as a transporter of calcium and phosphate, and is thus involved in the mineralization of bones (Figure 1, Table 2) [59]. Additionally, it interacts with insulin receptors, acts as a protective factor in severe inflammatory conditions, and is involved in atherosclerosis [59]. Serum fetuin-A was shown to be increased in obese or overweight PCOS patients in comparison to control women without PCOS, suggesting an association between serum fetuin-A and PCOS [60]. Moreover, studies have shown elevated fetuin-A levels in individuals with insulin resistance, with a positive association with Homeostatic Model Assessment for Insulin Resistance (HOMA-IR), glycated hemoglobin, low-density lipoprotein cholesterol, and body mass index (BMI), all markers of insulin resistance [61]. The underlying mechanism is the inhibition of the insulin receptor tyrosine kinase and toll-like receptor in the liver and muscle by fetuin-A, causing repression of insulin signaling and stimulation of inflammatory signaling pathways [60].

Furthermore, insulin resistance in the long term results in hyperinsulinemia, which has been linked with a surge in luteinizing hormone (LH) and an increase in androgen secretion from theca cells via ovarian insulin receptors [62]. Further studies are required to understand the effects of the underlying mechanisms on hormone levels.

### 4.2. Fetuin B

**Figure 2:**
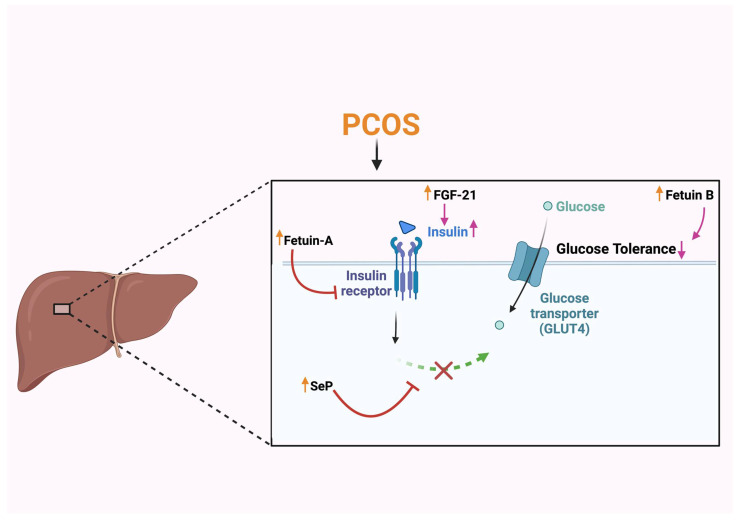
The role of hepatokines in the progression of PCOS. A schematic illustrating the elevation of hepatokines in PCOS. Fetuin-A inhibiting insulin tyrosine kinase receptor. FGF-21: fibroblast growth factor-21 increasing insulin levels. SeP: selenoprotein, inhibiting the insulin signaling pathway. Fetuin B reducing glucose tolerance. The elevated levels contribute to the development of insulin resistance, a key feature of PCOS.

Fetuin B is a hepatocyte-derived protein involved in glucose metabolism and is part of the cysteine protease inhibitor family (Figure 2) [63]. Studies have reported increased serum fetuin B in PCOS patients, with the suggestion that fetuin B could be a factor associated with NAFLD in PCOS through enhancing insulin resistance [64]. A study has shown that, in humans, the regulation of fetuin B is by steatosis. Additionally, fetuin B is elevated in type 2 diabetes and regulates glucose metabolism independently of insulin, which leads to impaired glucose tolerance [65]. An in vivo study was conducted by administering a physiological concentration of fetuin B to cultured muscle and hepatocytes, reducing insulin sensitivity [65]. In a study using a glucagon-like-peptide-1 receptor agonist (GLP-1RA) for six months in PCOS, there was a decrease in serum fetuin B levels, indicating the association between fetuin B and glucose metabolism [66]. This suggests that fetuin B is involved in the pathophysiology of PCOS through the metabolism of glucose.

### 4.3. Fibroblast Growth Factor 21

Fibroblast growth factor 21 (FGF-21) is a hepatokine that plays a vital role in the metabolism of glucose and lipids [67]. FGF-21 is significantly increased in PCOS patients, as reported by several studies [61], and this elevation occurs regardless of obesity [68]. Elevation of FGF-21 levels in PCOS women may result in increased circulating free fatty acids and insulin under metabolic stress.

Furthermore, FGF-21 has been shown to stimulate the release of adiponectin, improving glucose control and enhancing insulin sensitivity [69]. Studies have also shown increased FGF-21 concentrations in PCOS patients with NAFLD versus PCOS patients without NAFLD, implying the association between FGF-21, metabolic parameters, and PCOS [70]. However, some studies are contradictory, stating that circulatory concentrations of FGF-21 are independent of metabolic parameters in women with PCOS [71]. Therefore, further research is needed to clarify the underlying mechanism and importance of increased FGF-21 concentrations in PCOS women.

### 4.4. Hepassocin

Hepassocin (HPS) is a hepatokine which facilitates communication between hepatocytes and muscle cells [72]. HPS is associated with NAFLD and insulin resistance due to the increased expression of HPS in NAFLD [72]. The rise in HPS is caused by fat accumulation, resulting in impaired insulin signaling and ultimately leading to the development of type 2 diabetes mellitus. Additionally, studies have revealed higher HPS levels in obese PCOS patients versus non-obese PCOS patients [72]. An independent association between (HOMA-IR) and elevated HPS has been demonstrated, suggesting that the rise in HPS in obese PCOS patients may be due to IR [72], though further investigation is required to confirm that.

### 4.5. Selenoprotein

Selenoprotein (SeP) is one of the hepatokines that is involved in the metabolism of glucose in the body [73]. SeP is elevated in PCOS patients in comparison with healthy individuals. In those PCOS subjects with increased SeP, elevated levels of insulin, HOMA-IR, and testosterone were also found [73]. Another study showed that administering purified SeP caused impaired insulin signaling and dysregulated glucose metabolism in hepatocytes, leading to insulin resistance [74]. The elevated testosterone levels seen in PCOS appear to be associated with increased levels of SeP. SeP may be involved in the inflammation and oxidative stress that can accompany the hormonal imbalance in PCOS [75]. Overall, these hepatokines emerge as critical mediators in the complicated interplay between hormonal dysregulation, metabolic disturbances, and the development of PCOS-related comorbidities such as NAFLD, and potentially also in the progression of NAFLD. Further research is required to clarify the underlying mechanisms and to identify potential therapeutic targets of these hepatokines in the management of PCOS and their contribution to NAFLD.

**Table 2:** The role of selected hepatokines in the pathophysiology of PCOS and NAFLD.

Hepatokine	Role in PCOS	Role in NAFLD	Reference
Fetuin-A	Increased in obese or overweight PCOS patients. Fetuin-A can contribute to insulin resistance in PCOS patients by inhibition of the insulin signaling pathway and activation of inflammatory pathways.	Increased in patients with NAFLD, levels reduced after improvement of the disease. Fetuin-A is involved in steatosis of the liver and metabolic syndrome, not the underlying inflammatory process. Fetuin-A plays a role in the progression of nonalcoholic fatty liver (NAFL) to nonalcoholic steatohepatitis (NASH), indicating the association between Fetuin-A and steatosis.	[60,76,77]
Fetuin B	Elevated serum fetuin B in PCOS patients. Fetuin B plays a role in the development of insulin resistance; however, the mechanism is not fully understood.	Increased fetuin B in patients with NAFLD. Fetuin B is positively correlated to triglyceride (TG) levels, as well as hepatic steatosis.	[64,68,77]
Fibroblast growth factor- 21	Increased in patients with PCOS. FGF-21 has been shown to increase circulating free fatty acids, insulin, and adiponectin, improving glucose control and insulin sensitivity.	FGF-21 is elevated in patients with NAFLD and correlates with the severity of liver inflammation in NAFLD. FGF-21 is also increased in obesity, metabolic syndrome, and insulin resistance. Hence, it could be used as a biomarker for the diagnosis of metabolic disorders. FGF-21 is associated with markers of liver inflammation, such as M30 fragment (a caspase-cleaved fragment of cytokeratin 18) and plasminogen activator inhibitor-1 (PAI-1).	[68,69,78,79]
Hepassocin	Higher in obese PCOS patients. Further research is required to fully understand the mechanism.	High levels of HPS in patients with NAFLD. Increased expression of HPS causes upregulation of lipogenic proteins, ultimately leading to an increase in the accumulation of hepatic triglycerides (TGs). Upregulation of HPS stimulates inflammatory cytokines and liver injury.	[72,80]
Selenoprotein	Increased in PCOS patients. SeP disrupts the pathway of insulin signaling, resulting in decreased glucose uptake and increased gluconeogenesis.	SeP is elevated according to the severity of NAFLD. SeP is associated with the degree of liver fibrosis. Increased SeP promotes insulin resistance and oxidative damage to the liver, hence inducing liver inflammation.	[73,74,81]

## 5. Genetic Liver Involvement in PCOS Development

The genetic interplay between NAFLD and the development of PCOS requires clarification. A study utilizing bioinformatics to explore this connection using two datasets, GSE34526—PCOS and GSE63067—NAFLD, showed 52 differentially expressed genes (DEG) in the two datasets. From those, nine HUB genes were selected: TREM1, S100A9, FPR1, NCF2, FCER1G, CCR1, S100A12, MMP9, and IL1RN. Additionally, four miRNAs were identified that interacted to some degree with the nine HUB genes—miR-20a-5p, miR-129-2-3p, miR-124-3p, and miR-101-3p [8]—suggesting that the HUB genes are essential in promoting NAFLD and PCOS, and implying that these HUB genes could serve as potential biomarkers for diagnosis and monitoring of patients with NAFLD and/or PCOS.

### 5.1. Non-Coding RNA and Proprotein Convertase Subtilisin/Kexin Type 9 Inhibitor

**Figure 3:**
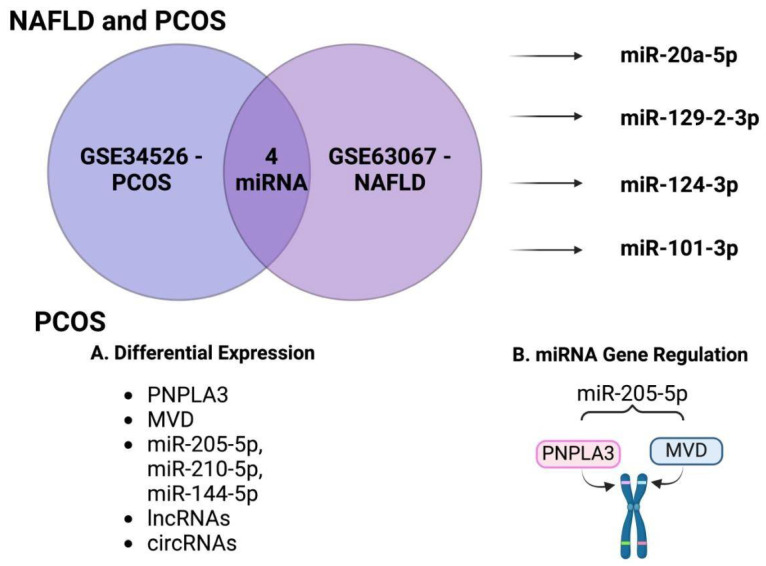
A bioinformatics dataset to determine genes involved in PCOS and NAFLD. A schematic demonstrates 9 HUB genes identified as significant and 4 miRNAs with substantial interactions with the HUB genes. (**A**) The genes PNPLA3 and MVD, miR-205-5p, miR-210-5p, miR-144-5p, lncRNAs, and circRNAs were all identified as differentially expressed in PCOS-specific datasets. (**B**) miR-205-5p modulates the genes PNPLA3 and MVD, essential for metabolism and ovarian steroidogenesis in PCOS. PNPLA3: patatin-like phospholipase domain-containing 3; MVD: mevalonate miphosphate mecarboxylase.

Bioinformatics was used to analyze six datasets to determine which miRNAs were differentially expressed in PCOS (Figure 3). Of the six datasets, GSE138572 and GSE168404 were identified due to the differential expression of several miRNAs [82]. This study concluded that mRNA from patatin-like phospholipase domain-containing 3 (PNPLA3) and mevalonate miphosphate mecarboxylase (MVD), and several miRNAs (hsa-miR-205-5p, hsa- miR-210-5p, has-and miR-144-5p), together with miRNA-associated lncRNAs and circRNAs, play critical roles in the initiation and progression of PCOS [82]. Furthermore, the role of proprotein convertase subtilisin/kexin type 9 (PCSK9) as a potential underlying mechanism in the pathogenesis of PCOS was highlighted. PCSK9 is an essential hepatocyte protein regulating low-density lipoprotein receptors (LDLRs) [82]. Patients with PCOS were found to have abnormally elevated levels of PCSK9, suggesting that this may result in the correlation of elevated low-density lipoprotein (LDL) levels in PCOS. This highlights the vital role of the liver, as hepatocytes are one of the most prominent synthesizers of PCSK9 [82]. This concept links obesity with the LDLR in the liver in subjects with PCOS. PCSK9 warrants further exploration as a potential mechanistic connection between PCOS and NAFLD. The potential benefit of PCSK9 inhibitors for NAFLD patients has been suggested; however, future research should focus on PCSK9 inhibitors and their therapeutic role in PCOS and NAFLD patients. PCSK9i can potentially benefit patients with either metabolic disorder as these medications could increase the clearance of LDL from the circulation, helping improve NAFLD symptoms while lowering the PCSK9 protein level commonly elevated in PCOS patients [83].

A preliminary finding reported the role of miRNA secreted from exosomes and obesity, insulin resistance, and PCOS [84]. Using mouse models, exosomal overexpression of miR-20b-5p and miR-106a-5p was found to inhibit adipocyte differentiation in 3T3-L1 cells [84]. These miRNAs are, therefore, potentially linked to hepatic lipid metabolism. The genetic interactions may be reflected in non-coding RNA expression with the miR-761- hepcidin/Gpx4 pathway that was investigated to determine, through monitoring ferritin, whether dysregulated iron levels would be present in PCOS and/or NAFLD [85]. No significant difference in ferritin levels was found between PCOS patients with and without NAFLD; however, increased ferritin levels were noted in both PCOS and NAFLD patients [72].

Furthermore, patients with both PCOS and liver damage not attributed to NAFLD had significantly higher ferritin levels relative to controls [85]. These findings align with results reported in a mouse model of PCOS [72]. Using hepcidin, a protein responsible for decreasing iron absorption from the gastrointestinal tract and preventing iron release from storage, researchers determined that miR-873-5p and miR-761 have opposite expression levels relative to hepcidin [85]. If these miRNAs are highly expressed and hepcidin is low, this could provide a potential mechanism for the increased ferritin and iron in individuals with NAFLD and PCOS. Further research is warranted to understand the possible mechanism and its contribution to disease.

### 5.2. Sex Hormone Binding Globulin

**Figure 4:**
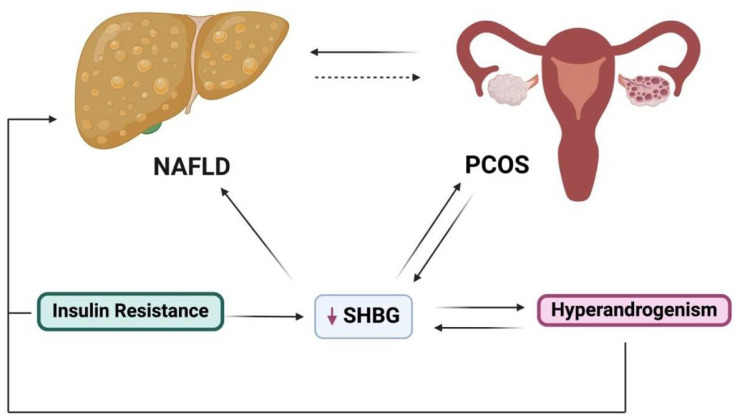
The association of SHBG with PCOS and NAFLD. Women with NAFLD and/or PCOS present with lower SHBG levels. PCOS is associated with hyperandrogenism and insulin resistance, which not only lowers SHBG levels but also contributes to an increased risk of developing NAFLD. SHBG: sex hormone binding globulin; PCOS: polycystic ovary syndrome; NAFLD: nonalcoholic fatty liver disease.

Sex hormone binding globulin (SHBG) is found to be decreased in both NAFLD and PCOS (Figure 4) [86]. These findings are further supported by a study investigating NAFLD patients and a separate study of PCOS patients [87,88]. One study reported that young women with biopsy-proven NAFLD and decreased levels of SHBG have a higher likelihood of developing PCOS, further supporting the possible bidirectional relationship, as well as the potential use of SHBG as an accurate biomarker for metabolic disorders such as NAFLD and PCOS [89]. Chang et al. proposed the liver–ovarian axis with reference to SHBG levels and their relationship to PCOS and NAFLD, suggesting that lower SHBG levels are associated with PCOS while also being associated with NAFLD and insulin resistance in women with PCOS [90]. Whether lower SHBG contributes to the development of NAFLD and thus leads to PCOS or whether lower SHBG levels result from NAFLD, which dysregulates the reproductive system and contributes to PCOS, requires further clarification. However, current knowledge suggests a significant interplay between SHBG levels and NAFLD and PCOS. In PCOS women, it has been shown that hyperandrogenemia is exacerbated by insulin resistance, thereby leading to an increase in androgen production in the ovaries as well as a decrease in sex hormone binding globulin (SHBG), resulting in excess circulation of free androgens due to an increase in bioavailability [91,92].

Additionally, studies have indicated that a reduction in SHBG levels contributes to the development of NAFLD, making it a sensitive biomarker of NAFLD [86], and SHBG is suggested as a surrogate marker for insulin resistance in PCOS [93]. A longitudinal cohort study that investigated the association between NAFLD and circulating SHBG levels showed an inverse association between SHBG levels and the severity of steatosis and portal inflammation. This suggests the existence of a relationship between NAFLD and adipocyte–hepatocyte inflammation since portal inflammation has been known to denote fatty liver disease [94]. Furthermore, a longitudinal population-based cohort study that analyzed 63,000 women diagnosed with PCOS against matched controls (incorporating a total of 121,000 participants) showed that anovulation and androgen excess independently increased the risk of NAFLD development [95]. Research has shown sexual dimorphism in liver metabolism, where androgen levels in men prevent hepatic fat accumulation. In contrast, higher androgens in women increase the risk of NAFLD in PCOS [96]. Therefore, understanding the pathophysiology, including the development and progression of NAFLD, is critical, especially due to the gender differences in the clinical presentation of NAFLD [96].

### 5.3. Cannabinoid 1 Receptor

Increased cannabinoid 1 receptor (CNR1) expression is found in liver diseases such as NAFLD. If hepatocyte CNR1 is downregulated, toxic effects are reduced, resulting in reduced liver damage [97]. This reduced toxin damage is effected via modulation of the proinflammatory NF-kB signaling pathway [97], and it is well recognized that the pathogenesis of PCOS is associated with low-grade chronic inflammation [98]. In a study of 445 women with PCOS, regardless of body habitus, testosterone, and hormonal parameters, upregulated NF-kB signaling was found compared to the non-PCOS control group, highlighting the significance of inflammation in both PCOS and NAFLD [98]. Another study reported that the polymorphism for allele G of rs806381 was associated with PCOS patients who developed NAFLD [99]. A further report showed that within their sample of 174 women with PCOS versus 125 control women without PCOS, there was a three-fold increased risk for the GG genotype of polymorphism rs12720071, and there was an eight-fold increased risk for women expressing the genotype CC polymorphism associated with rs806368 [100]. Thus, genetics plays a significant role in the predisposition of an individual to NAFLD and/or PCOS while further highlighting the interconnectedness of the diseases. Further research is needed to explore the genetic relationship, especially the reverse circumstance of women with NAFLD developing PCOS. However, current evidence is suggestive of a link, warranting further exploration of the relationship between CNR1 upregulation and the development of these two disorders.

### 5.4. Patatin-like Phospholipase Domain-Containing 3

The patatin-like phospholipase domain-containing 3 gene (PNPLA3) was studied with the aim of determining the frequency of the polymorphism rs738409 in patients with PCOS and its role in NAFLD risk and disease severity [101]; this polymorphism was found to increase the prevalence of NAFLD in women with PCOS, including both those heterozygous and homozygous for the polymorphism [101]. For women with NAFLD and polymorphisms affecting PNPLA3, a connection to PCOS development may exist, and PNPLA3 polymorphisms may act synergistically to increase NAFLD disease risk [101]. This synergistic effect could underpin a reverse connection between these two metabolic diseases, with NAFLD leading to PCOS. Further PNPLA3 interactions relative to NAFLD were reported in another study that determined that the I148M mutant of PNPLA disrupted ubiquitylation and negatively impacted the degradation of PNPLA3. Consequently, a rise in hepatocyte accumulation of triglycerides and poly-unsaturated fatty acids (PUFAs) occurred, resulting in a fatty liver phenotype [102].

Specifically, patients with the variant 148M/M had much higher liver fat content, lower serum fasting triglyceride levels, and less insulin resistance [102]. These data provide a link between PNPLA3, NAFLD, and PCOS. PNPLA3 could be further explored as a potential biomarker for NAFLD patients developing PCOS.

### 5.5. Cytochrome P450

**Figure 5:**
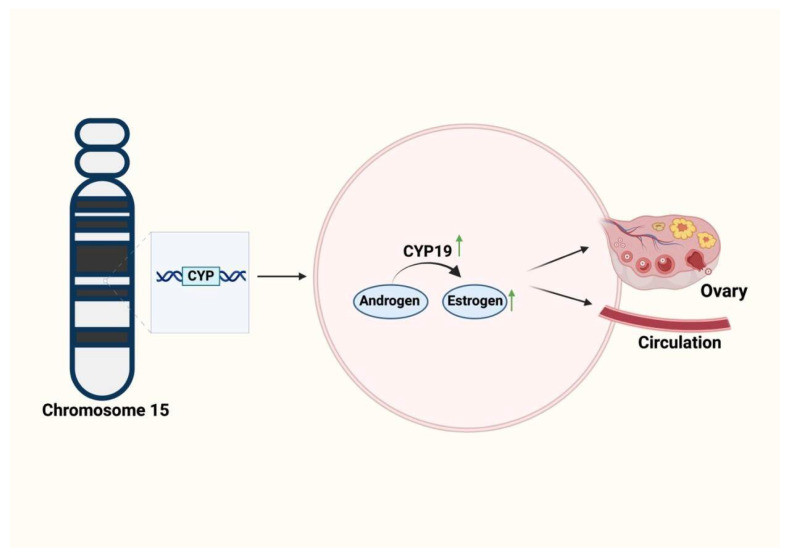
The effects of CYP19A1 on the development of PCOS. A schematic to illustrate how upregulation of CYP19A1 in PCOS leads to an increase in estrogen levels.

Cytochrome P450 (CYP450) is a superfamily of proteins responsible for producing an array of metabolic enzymes required for homeostatic maintenance. When these enzymes are disrupted, metabolic diseases such as NAFLD and PCOS can arise. CYP11a, CYP21, CYP17, and CYP19 are key genes associated with dysregulation of androgen production (Table 3) [103]. In a study of Pakistani families, it was found that family 5 had a significant PCOS marker, D15S519, at chromosome 15 [103]. This marker originates from CYP11a, associated with PCOS etiology [104]. Upregulation of CYP19A1 affects aromatase activity and promotes estrogen production (Figure 5) [105]; CYP19A1 is located on chromosome 15 at 15q21.2 [105] and produces an enzyme responsible for converting testosterone to estrogen [106]. The current literature highlights an established relationship between certain cytochrome families and PCOS, as well as a connection between cytochrome families and metabolism [107]. Thus, there is evidence to explore further the CYP family’s role in PCOS and/or NAFLD. When these CYP genes are impaired, aberrations of glucose and lipid metabolism occur, and the liver is a key player in both [107]. This evidence indicates the effect of chromosome 15 genes on estrogen levels, potentially driving PCOS development and abnormal lipid metabolism and contributing to NAFLD. To further explore this hypothesis, CYP4A14’s role in the development of NAFLD was investigated [108], and it was found that upregulation of CYP4A14 contributed to lipid accumulation within the liver, whereas, when CYP4A14 was ablated, the liver damage attributable to NAFLD was attenuated [108]. No CYP connection between NAFLD and PCOS has yet been firmly established. Future research could explore the potential impact of CYP genes on the development of these two metabolic disorders.

### 5.6. Liver Clock Genes

**Figure 6:**
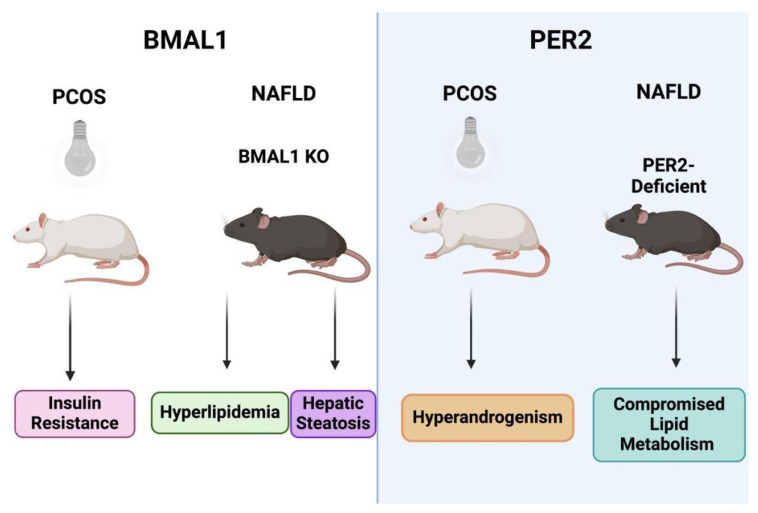
The role of two key liver clock genes, BMAL and PER2. BMAL1, a liver clock gene, is a key modulator of metabolic function. The PCOS rat model exposed to constant darkness developed decreased levels of BMAL1, resulting in insulin resistance. The NAFLD BMAL1 knock-out mouse model developed hyperlipidemia and hepatic steatosis. PER2, a liver clock gene, is also a key modulator of metabolic function. The PCOS rat model exposed to constant darkness developed decreased levels of PER2, resulting in hyperandrogenism from the excessive excretion of Insulin-Like Growth Factor-Binding Protein 4 and SHBG. The NAFLD PER2-deficient mouse model exhibited dyslipidemia.

Circadian clock genes are responsible for maintaining homeostasis of every physiological process within the body. These genes are influenced by light/dark cycles and can be upregulated or downregulated depending on the body’s metabolism and the required response. A study highlighted the importance of the liver clock genes *BMAL1*, *PER1*, and *PER2* in PCOS (Figure 6) [112]; when rats were exposed to constant darkness, their liver clock genes became desynchronized with the environment, causing a decrease in BMAL1 levels, which promoted insulin resistance [112]. In addition, the constant darkness treatment group demonstrated decreased levels of both PER1 and PER2, which promoted androgen excess due to excessive production of Insulin-Like Growth Factor-Binding Protein 4 and SHBG from the liver. Both insulin resistance and androgen excess are well-known features of PCOS [112].

Regarding NAFLD, a study demonstrated the importance of circadian clock genes for the maintenance of homeostatic liver function [113] in a mouse model where circadian clocks and feeding times were shown to play a key role in maintaining hepatic triglyceride levels. Increased hepatic triglyceride levels can lead to NAFLD and obesity, and this further supports the connection between NAFLD and PCOS development [113]. Specifically, BMAL1 knock-out mice developed hyperlipidemia and hepatic steatosis, while PER2-deficient mice demonstrated abnormal lipid metabolism [114,115], emphasizing the importance of liver clock genes in the development of both NAFLD and PCOS.

A study explored the function of the nuclear receptor subfamily 1 group D (REV-ERB) in PCOS, as both the alpha and beta nuclear receptors within the REV-ERB family help control circadian clock functioning [116]. The in vitro analysis demonstrated that when REV-ERB alpha and beta were overexpressed using the agonist stenabolic (SR9009), the peroxisome proliferator-activated receptor-γ coactivator (PGC-1alpha), nuclear respiratory factor 1 (NRF1), and mitochondrial transcription factor A (TFAM) genes were all promoted [116]. Each of these three genes is critical for mitochondrial biosynthesis pathways. Not only were mitochondrial genes upregulated, but there was also inhibition of apoptosis within ovarian granulosa cells and increased proliferation, further contributing to the PCOS disease model [116]. Interestingly, using the same model in vivo to explore the effects of overexpression of REV-ERB using the SR9009 agonist and its impact on the liver [117], improvement in liver histology was noted, with a reduction in fibrosis and inflammation [117]. Therefore, the modulation of REV-ERBs could effectively modulate the interplay between NAFLD and PCOS.

## 6. Health Implications and Future Directions

This review article has explored existing knowledge about the association between PCOS and liver function. The correlation between insulin signaling impairment in PCOS, the factors causing insulin resistance in patients, and the association between NAFLD and PCOS have been explored; further, the inflammatory markers elevated in PCOS that are interconnected to liver function, the metabolic effects of both PCOS and NAFLD, and the role that genetics plays in both conditions have been discussed.

Future research could investigate the following:

- Determination of the underlying mechanism of fetuin-A and SeP in NAFLD;
- Further exploration of the underlying mechanism of HPS in PCOS and NAFLD;
- Clarification of the genetic links between PCOS and NAFLD and the use of genetic links to guide therapeutic strategies;
- Identification of the underlying mechanisms contributing to the crosstalk and bidirectional relationship between NAFLD and PCOS;
- Confirmation of whether PCOS causes liver dysfunction or liver dysfunction causes PCOS;
- Identification of the association between NAFLD and PCOS in relation to hepatokines and their use as biomarkers and/or therapeutic targets;
- Long-term follow-up studies to monitor the optimal management of PCOS patients with NAFLD.

## 7. Conclusions

PCOS and NAFLD are both common disorders, each having a distinct morbidity and potential mortality that require effective therapeutic strategies. There is an established literature showing that women with PCOS are at higher risk of developing NAFLD; however, significantly less research has explored the converse relationship. Studies into lipid metabolism, lipotoxicity, and oxidative stress have demonstrated preliminary connections between these two metabolic disorders. Lipotoxicity causes androgen excess and contributes pathogenically to both NAFLD and PCOS, while oxidative stress increases ROS, contributing to chronic inflammation, a symptom common to both disorders. The hepatokines fetuin-A and SeP appear to have importance in PCOS pathophysiology, though less is known about their role in NAFLD.

From a genetic perspective, there is a significant heritability for both metabolic diseases, with additional evidence to support an increased risk of developing PCOS in genetically predicted NAFLD. Through the exploration of specific genes, miRNAs, and proteins using bioinformatics, in vivo models, and in vitro models, there is evidence for crosstalk between NAFLD and PCOS. SHBG levels are reduced in NAFLD and PCOS and could serve as a useful biomarker for diagnosis and disease monitoring. CYP450 family proteins, notably CYP11a and CYP19A1, impact estrogen levels and lipid metabolism. Liver clock genes *BMAL1* and *PER2* are similarly reduced in animal models for both PCOS and NAFLD, emphasizing the commonality between these metabolic disorders.

Furthermore, PCSK9i demonstrated utility as a treatment for both disorders, increasing the clearance of LDL from the circulation and improving NAFLD symptoms whilst decreasing the higher-than-normal levels of PCSK9 in patients with PCOS. Genetic linkage between NAFLD and PCOS is likely. However, further research is needed to elucidate these links fully and to determine which underlying mechanisms are contributing to the crosstalk and bidirectional relationship between NAFLD and PCOS. Only then will it be possible to definitively answer the question as to how liver dysfunction contributes to PCOS and how PCOS contributes to liver dysfunction.

## Figures and Tables

**Table 3:** Key genes of the CYP450 family involved in the progression of PCOS or NAFLD.

CYP450 Family	Disease Implication	Location	References
CYP11a	PCOS dysregulated androgen production	Marker D15S519, chromosome 15	[104]
CYP21	PCOS dysregulated androgen production	Chromosome 6	[103,109]
CYP17	PCOS dysregulated androgen production	Chromosome 10	[103,110]
CYP19A1	PCOS, altered aromatase activity	15q21.2, chromosome 15	[105]
CYP4A14	NAFLD, lipid accumulation	Chromosome 4	[108,111]

## Data Availability

No novel data were generated in the writing of this review article.

## Abbreviations

PCOS	Polycystic ovary syndrome
NIH	National Institutes of Health
AES	Androgen Excess Society
CLD	Chronic liver disease
NAFLD	Nonalcoholic fatty liver disease
MR	Mendelian randomization
NASH	Nonalcoholic steatohepatitis
SHBG	Sex hormone binding globulin
OS	Oxidative stress
ROS	Reactive oxygen species
GCs	Granulosa cells
FF	Follicular fluid
IR	Insulin resistance
SOC3	Suppressor of cytokine signaling 3
CRP	C-reactive protein
LH	Luteinizing hormone
SNPs	Single-nucleotide polymorphisms
BMI	Body mass index
FGF-21	Fibroblast growth factor 21
HPS	Hepassocin
HOMA-IR	Homeostatic Model Assessment for Insulin Resistance
PCSK9i	Proprotein convertase subtilisin/kexin type 9 inhibitor
DEG	Differentially expressed genes
LDLR	Low-density lipoprotein receptors
MAFLD	Metabolic-associated fatty liver disease
PNPLA3	Patatin-like phospholipase domain-containing three genes
GLP-1	Glucagon-like peptide-1
MI	Myo-inositol
GLP- 1RA	Glucagon-like peptide-1 receptor agonist
DCI	D-chiro-inositol
OCs	Oral contraceptives
DHT	Dihydrotestosterone
SiRNA	Small interfering RNA
MiRNA	MicroRNA
LncRNA	Long non-coding RNA
CircRNA	Circular RNA
